# Reliance into Action: Understanding EMA Documents to Streamline Reliance for Marketing Authorization Applications

**DOI:** 10.1007/s43441-025-00824-9

**Published:** 2025-06-28

**Authors:** Isabelle Colmagne Poulard, Susanne Ausborn, Martin Harvey Allchurch, Victoria Palmi, Alberto Ganan, Angelika Joos, Andrew Deavin, Corentin Beauchesne, Priti Shah, Jyothsna Krishnan, Chaima Askri

**Affiliations:** 1 Merck, 1 route de Crassier, Eysins, 1262 Switzerland; 2 Roche, Grenzacher Strasse 124, Basel, 4070 Switzerland; 3 European Medicines Agency (EMA), Domenico Scarlattilaan 6, Amsterdam, 1083 HS The Netherlands; 4 MSD, Boulevard du Souverain 25, Brussels, 1170 Belgium; 5 GSK, 20 Avenue Fleming, Wavre, 1300 Belgium; 6 Alexion, Av. Diagonal, 615, Les Corts, Barcelona, 08028 Spain; 7 Astra Zeneca, City House, Hills Rd, Cambridge, UK; 8 European Federation of Pharmaceutical Industries and Associations (EFPIA), Neo Building, Rue Montoyer 51, box 3, Bruxelles, 1000 Belgium; 9 Sartorius, Am Flughafen 16, 79108 Freiburg, Germany

**Keywords:** EMA, Manufacturing authorization application, Reliance, Regulatory convergence, CPP/eCPP

## Abstract

According to data gathered from across EU pharmaceutical trade associations, the European Medicines Agency (EMA) is one of the most frequently selected reference regulatory authority (RRA) for reliance procedures due its transparency, detailed decision-making reflected on its public assessment reports and easy access to information related to the assessment of medicines. This review discusses the implementation of unilateral reliance pathways for marketing authorization applications (MAAs) based on EMA assessment. The EMA established a focus group dedicated to reliance in 2022, with the primary aim of understanding the opportunities and hurdles encountered by European industry when using the EMA as RRA in global regulatory filings. A survey conducted among industry stakeholders revealed significant benefits of reliance pathways, including reduced approval timelines and decreased inquiries from relying authorities. However, the survey also highlighted persistent hurdles that hinder the benefits of unilateral reliance pathway, such as variability in documentation requirements and a lack of consistency across the documents requested by each national regulatory authority (NRA) when relying on EMA assessment. The findings highlight the need for collaboration between regulatory authorities and industry to streamline reliance processes and data requirements to make informed reliance decisions ultimately improving global access to safe, effective, and quality-assured medical products.

**Supplementary Information:**

The online version contains supplementary material available at 10.1007/s43441-025-00824-9.

## Introduction

In today’s globalized world, ensuring authorities around the world have a regulatory toolkit of regulatory sustainable processes which enable fast and equitable access to medical products is more critical than ever. One of those regulatory processes is Reliance, defined by the World Health Organization (WHO TRS 1033, Annex 10) [1] as follows: “*The act whereby the regulatory authority in one jurisdiction takes into account and gives significant weight to assessments performed by another regulatory authority or trusted institution*, *or to any other authoritative information*, *in reaching its own decision*.” However, reliance does not imply out-sourcing a relying agency’s decision-making authority or responsibility. In applying reliance in daily practice, national regulatory authorities (NRAs) maintain independence, sovereignty and accountability for their regulatory decision-making.

Reliance pathways bring benefits to patients, government and industry, through facilitating and accelerating access to quality-assured, effective, and safe medicinal products while saving resources and reducing duplication of regulatory efforts [1]. Over recent decades, different reliance pathways (e.g. collaborative pathways, work-sharing pathways, mutual recognition or unilateral reliance) have been implemented around the world.

The European regulatory system is based on reliance and recognition of decision among the different member states in the European Union (EU). With more than 30 years of experience, the European Medicines Agency (EMA) plays a fundamental role in facilitating the implementation of reliance worldwide through multilateral cooperation initiatives such as EU-M4all [2] WHO SRA-CRP [3] as well as through unilateral reliance [1] as reference regulatory authority (RRA) for other relying authorities.

The EMA Focus Group on reliance was created in 2022 to promote a multi-stakeholder discussion on issues related to the use of EMA as RRA in global filings, outline the current challenges and suggest practical recommendations and actions to facilitate reliance pathways more effectively. The Focus Group comprises members of the EMA International Affairs Department as well as EMA Committees and Quality Assurance Department, in addition to representation of industry experts from EU trade associations in line with EMA stakeholders’ engagement framework [4].

By examining the findings from a survey conducted among industry stakeholders in 2023, this review provides recommendations for implementing reliance more efficiently when using EMA outputs. This survey and related paper refer to reliance based on EMA as RRA also called unilateral reliance as defined by WHO [1]“when a country chooses to rely on an assessment from another country unilaterally and without reciprocity”.

## Method

A global survey was conducted between 7 March and 5 April 2023 with members of the EU pharmaceutical industry to understand the current use of EMA/EEA (European Economic Area) national competent authorities as RRA in reliance pathways for regulatory assessment of new marketing authorization applications (MAAs) or life cycle management (CMC post-approval changes, new indications, labeling variations (safety/efficacy) or line extensions).

EU trade associations members (EFPIA, Europabio, Vaccines Europe, Eucope and Medicines for Europe) were surveyed to gather input on reliance pathways that had been applied by companies within a two-year time period (between March 2021 and April 2023) for 70 countries worldwide in all regions excluding China and North America. This study thus focused on APAC (Asia Pacific), LATAM (Latin America), MEA (Middle East Africa) and EU countries which have been expecting/requesting reference country approval already at submission for national approval. Full list of countries can be found in Annex 2.

Forty-two companies (one response per company) provided anonymous responses related to qualitative and quantitative use of reliance based on EMA as RRA, reliance based on EU/EEA national competent authorities, as well as general questions related to opportunities, hurdles and improvement opportunities when using reliance. The second part of the survey quantitatively and qualitatively mapped the EMA output documents that are currently used as reliance tools for MAAs based on a pre-defined questionnaire.

In order to avoid biases and potential misinterpretation, it was decided only to consider survey responses on the use of reliance in a particular country if consistently mentioned by at least 5 company responses. Although not part of the initial scope of the survey, five respondents reported on Swissmedic and MHRA UK as RRA.

The full questionnaire can be found in Annex 1.

## Results

### Quantitative Use of Unilateral Reliance Based on EMA as Regulatory Reference Agency

From a total of 70 countries applying a form of reliance globally, companies reported the use of reliance based on EMA as RRA in 26 countries for MAA (37%), in 21 countries (30%) for new indications, labeling variations (efficacy/safety) or line extensions and in 16 countries (23%) for CMC post-approval changes.

The survey revealed that the majority of the 42 companies (71%) had used EMA as RRA across the different regulatory submission spectrum (from initial MAA to life cycle management). When analyzing the data in more detail, 17 responses highlighted the selection of EMA as RRA only (40%), 13 used either EMA and EU/EEA national competent authorities or Switzerland/UK (31%) and 5 used only EU/EEA national competent authorities (12%). The remaining 7 respondents (17%) did not report use of any form of reliance based on EMA or EU/EEA national competent authority(ies).

Companies reported that Germany, Italy, Netherlands, Spain and Austria were the EU/EEA national competent authorities most frequently used as RRAs. In addition to the EU/EEA national competent authority group, Swissmedic and MHRA UK were also reported in 5 instances.

### General Benefits and Potential Hurdles of Using Reliance

Companies were also asked to rank the perceived general benefits gained when using reliance as well as potential hurdles which need to be addressed to ensure its broader use (see Tables 1 and 2).

**Table 1:** Benefits of using reliance

	% of positive responses	Number of responses
Reduction of timelines to approval	95%	40
Reduction of number of questions from the relying agency	86%	36
Aligned PI (Product Information)	67%	28
Predictable review/approval timelines	64%	27
Reduction of country specific requirements and/or harmonization with SRA	48%	20
Capacity building (review and/or resources) amongst regulators	41%	17
Perceived reduced resource requirements for industry	41%	17
Other	2%	1

**Table 2:** Perceived hurdles when using reliance

	% of positive responses	Number of responses
Additional administrative requirements/documents including local M1 and other local documents	66%	27
Unredacted assessment report	54%	22
No clear reliance guideline or reliance not practiced	51%	21
Strict interpretation of product sameness	44%	18
No regulatory framework allowing reliance	41%	17
No Confidentiality Agreement or Memorandum of Understanding between reference authority and relying authority	34%	14
No clear understanding of reliance definition or no acceleration of timelines	24%	10
Not enough Reference Authorities to apply reliance or restricted scope	17%	7
Long submission lag time	15%	6
Difficulties in accepting eCPP	7%	3
None of the above or other	2%	1

In line with previous guidance [1] and publications [5–8] addressing perceived and tangible benefits and challenges in the implementation of reliance, the main benefit of reliance resides in faster availability of medicines for patients (through both reduced timelines to approval and gap between first approval and submission in LMIC countries). In addition, leveraging information from RRAs may enhance predictability and harmonization (reduction in the number of questions, aligned product information, predictable review timelines when clear reliance procedure requirements and timelines are available). Ultimately this helps build regulatory capacity through improved knowledge and experience.

Hurdles for selecting some reliance pathways may include higher administrative burden– mainly due to redundant or additional documentation requested, absence of clear guidance, lack of reliance documentation availability or legal barriers to be overcome (provision of unredacted assessment reports, lack of legal or regulatory framework allowing reliance, absence of confidentiality agreements/Memorandum of Understanding for sharing information), strict interpretation of product sameness beyond WHO definition [1] (product maybe the same but dossier may contain some differences to be justified [9]).

### Qualitative Use of Reliance Based on EMA as Regulatory Reference Agency

**Fig. 1:**
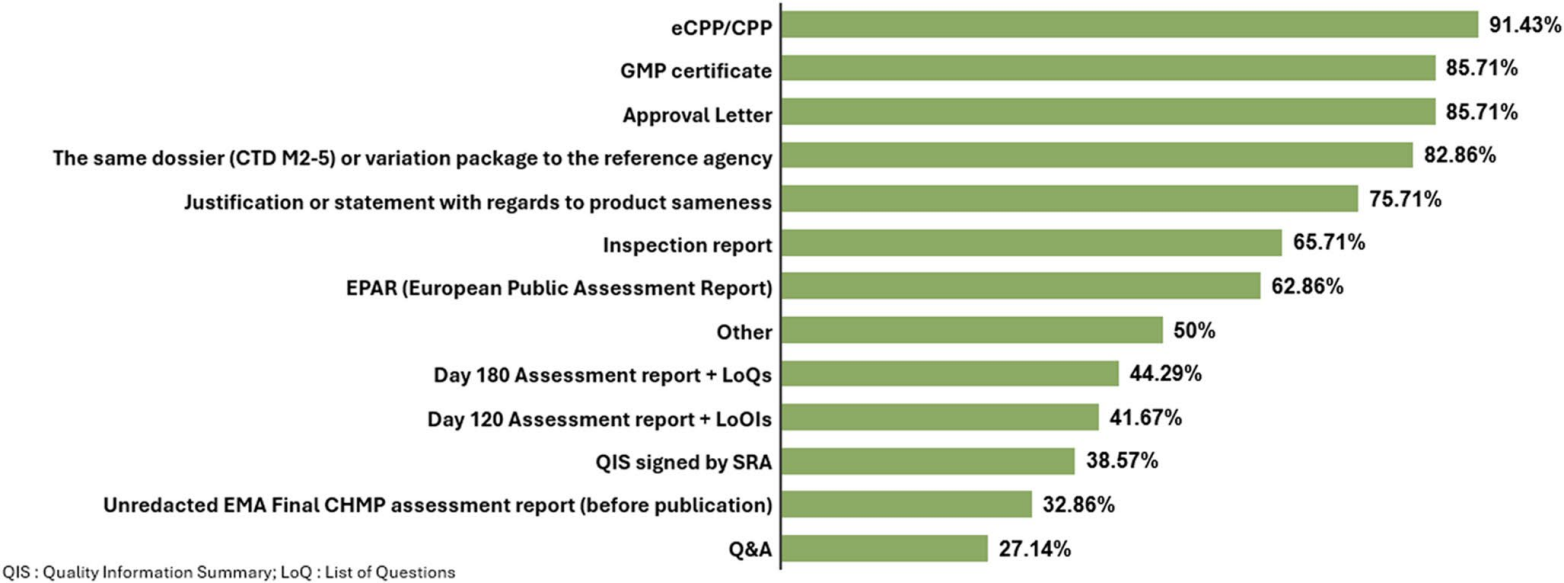
Documents provided in the application of reliance based on EMA as RRA

To better understand which EMA documents companies provide to support the use of reliance by NRAs, respondents had to select from a list, documents that might be used by each relying NRA. The list comprised thirteen documents selected based on companies’ experience of the most commonly requested information. These were: Approval Letter, CPP or eCPP (Certificate of Pharmaceutical Product in paper or electronic), EPAR (European Public Assessment Report), EMA Final CHMP (Committee for Medicinal Products for Human use) assessment report (before publication in the EPAR), Day 120 Assessment report and List of Questions (LoQ), Day 180 Assessment report and List of Outstanding Issues (LoOIs), GMP (Good Manufacturing Practice) certificate, Inspection report, Questions & Answers related to submission, Quality Information Summary (QIS) signed by SRA (Stringent Regulatory Authority), as well as “any other documents”. Figure 1 shows which documents were listed at least once out of the proposed 13 pre-listed documents (Fig. 1).

The most frequently provided document was the CPP (or eCPP when allowed) reported in 92% of responses, followed by use of GMP certificate or Approval letter (86%). Also frequently submitted to the relying authority (83%) was the same initial Marketing Authorization Application CTD (ICH Common Technical Document) dossier or modules for variation packages as originally submitted, as well as a justification or statement to confirm sameness [1] between the original dossier and the one submitted to the relying authority (76%).

## Discussion

Some forms of reliance (e.g., based on the use of CPPs) have been applied globally for decades. For MAAs, reliance is often used by a given NRA either as an abridged or verification procedure which takes into consideration the assessments from a trusted RRA like US FDA and/or EMA. Over time, many countries have implemented their own national reliance pathways, resulting in a very diverse regulatory landscape with different scope, procedures and requirements [10–12].

### EMA as Regulatory Reference Authority (RRA)

**Fig. 2:**
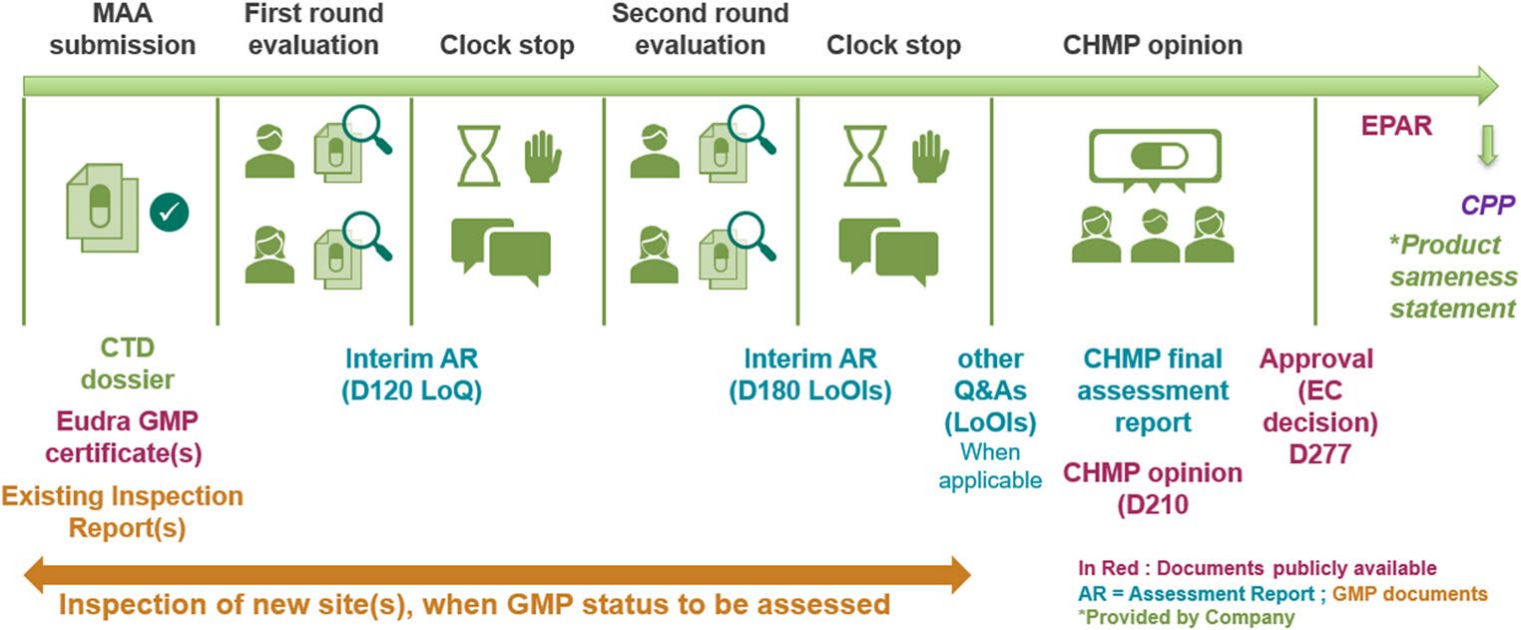
Documents generated during the EMA Centralised Procedure (MAA)* *except ‘Description of Differences’ as Company Statement and CPP generated upon request by EMA outside of MAA centralised procedure

As per WHO Good Reliance Practices [1] EMA supports reliance as a general principle to make the best use of available resources and expertise. This study was helpful to gain some insight from industry on how many NRAs around the world are leveraging EMA as RRA and are provided with EMA assessment outcomes. Based on the results obtained, it can be concluded that EMA has been widely used as RRA around the globe (by 71% of the 70 countries in scope of the survey). This can be explained by the fact that the EMA strives towards being as transparent and comprehensive as possible about how it comes to its decisions: a high number of documents (outputs) from the centralised procedure are publicly available on the EMA or EC (European Commission) website or, when not publicly available, can be obtained from EMA (or the applicant) through an appropriate procedure (see Fig. 2). In order to further understand the use of EMA assessment outputs for implementation of reliance, since 2024, MAH/Applicants have been invited to inform EMA about which EMA documents are shared with regulators outside the EU for reliance on a periodic basis [13].

### Opportunities and Hurdles when Using Reliance for MAAs

In line with the CIRS 2021 study [6] and 2024 workshop [7] companies highlighted benefits when using reliance efficiently (see Table 1), with demonstrated timelines to approval shorter than the standard timelines. Companies also noted a possible gain coming from reduction of the number of questions from the relying authorities, thus offering patients and healthcare providers timely access to safe, effective and quality medicinal products. However, some potential hurdles remain related to the level of documentation required with no clear guidance on implementation of reliance or additional local requirements for Module 1 (and 3) required as well as specific issues related to access to full assessment reports (see Table 2).

Differences between requirements across reliance pathways present a challenge to applicants looking to submit a single dossier globally. When analyzing the documents (outputs from the centralised procedure as depicted in Fig. 2) that are used in the context of reliance for MAAs, there was no consistency in the number and types of documents submitted. According to the survey results, the full range of listed documents were required by some relying authorities (see Fig. 1). While in most cases the CPP and approval letter were required, 83% of countries requested more documents (not counting additional specific CTD Module 1 and 3 requirements).

### Analysis of EMA Centralised Procedure (for MAA) Outputs that Can Be Used as Reliance Tools

**Fig. 3:**
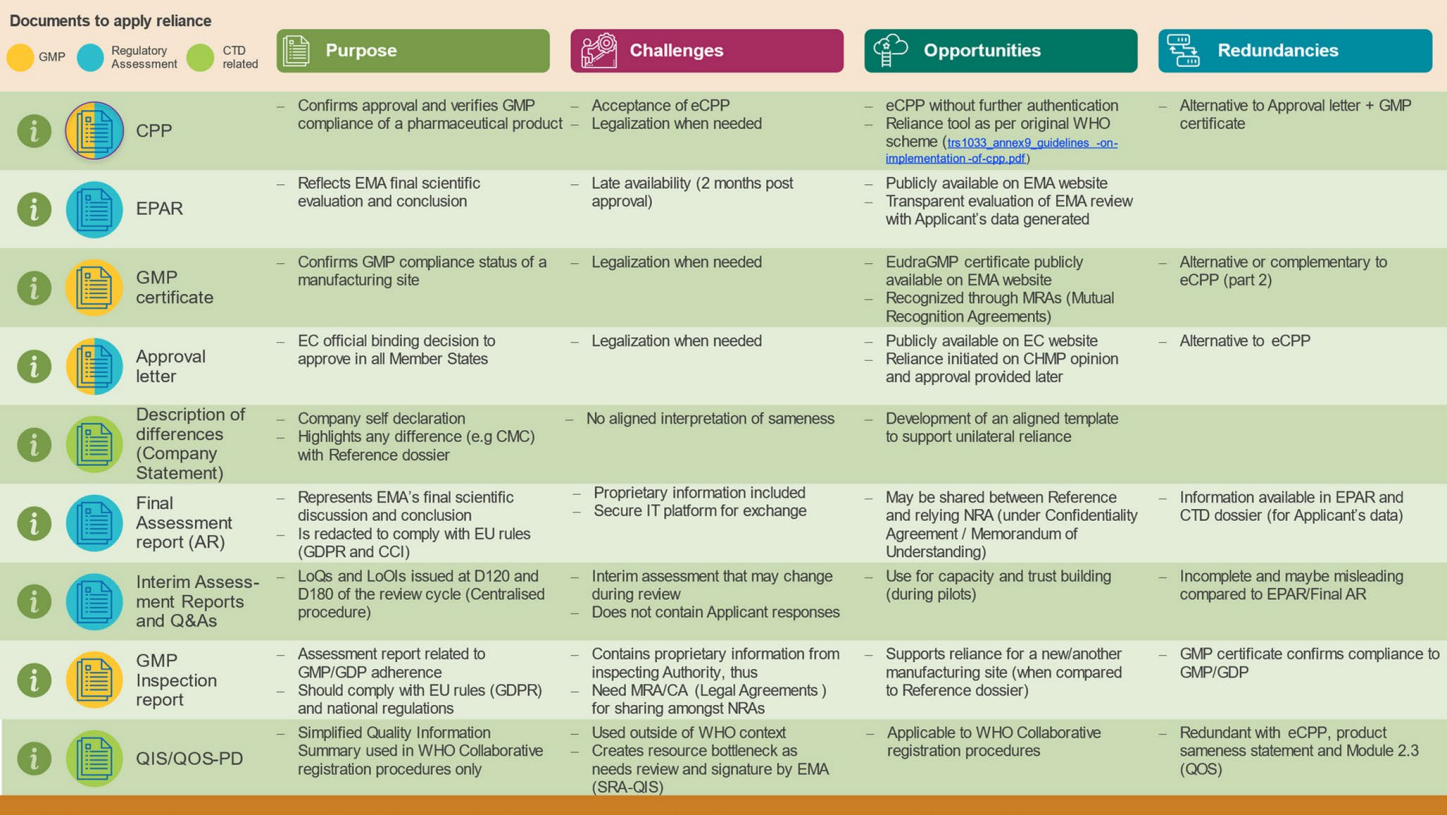
EMA Reliance tools*. *except Product Sameness Statement and QIS/QOS-PD as WHO Template

Understanding the different documents that EMA produces is essential to understand what outputs are key or complementary for use in regulatory reliance and which ones might be considered redundant as they do not add any additional information from a scientific perspective. Therefore, a more thorough analysis of the documents currently provided to relying authorities by the applicant or marketing authorization holder (MAH) was conducted to identify which documents should be considered as primary reliance tools, which are providing supportive information and which would be duplicative. A summary of the purpose, challenges and opportunities as well as redundancies between documents is presented in Fig. 3 (note: regulatory assessment related in blue, GMP related in yellow or CTD package related in green).

When selecting documents required to implement reliance, NRAs are encouraged to consider the context of use (WHO collaborative pathway, unilateral reliance pathway, capacity building exercise), purpose, challenges and opportunities. This is important to avoid duplication of requirements and enable a reliance process based on sufficient and appropriate documentation provided by the applicant or MAH, thus supporting an informed decision-making on the approval of the medicine (referred to as “informed reliance [14]”).

### Key Considerations and Recommendations Related To Reliance Implementation for MAAs

**Fig. 4:**
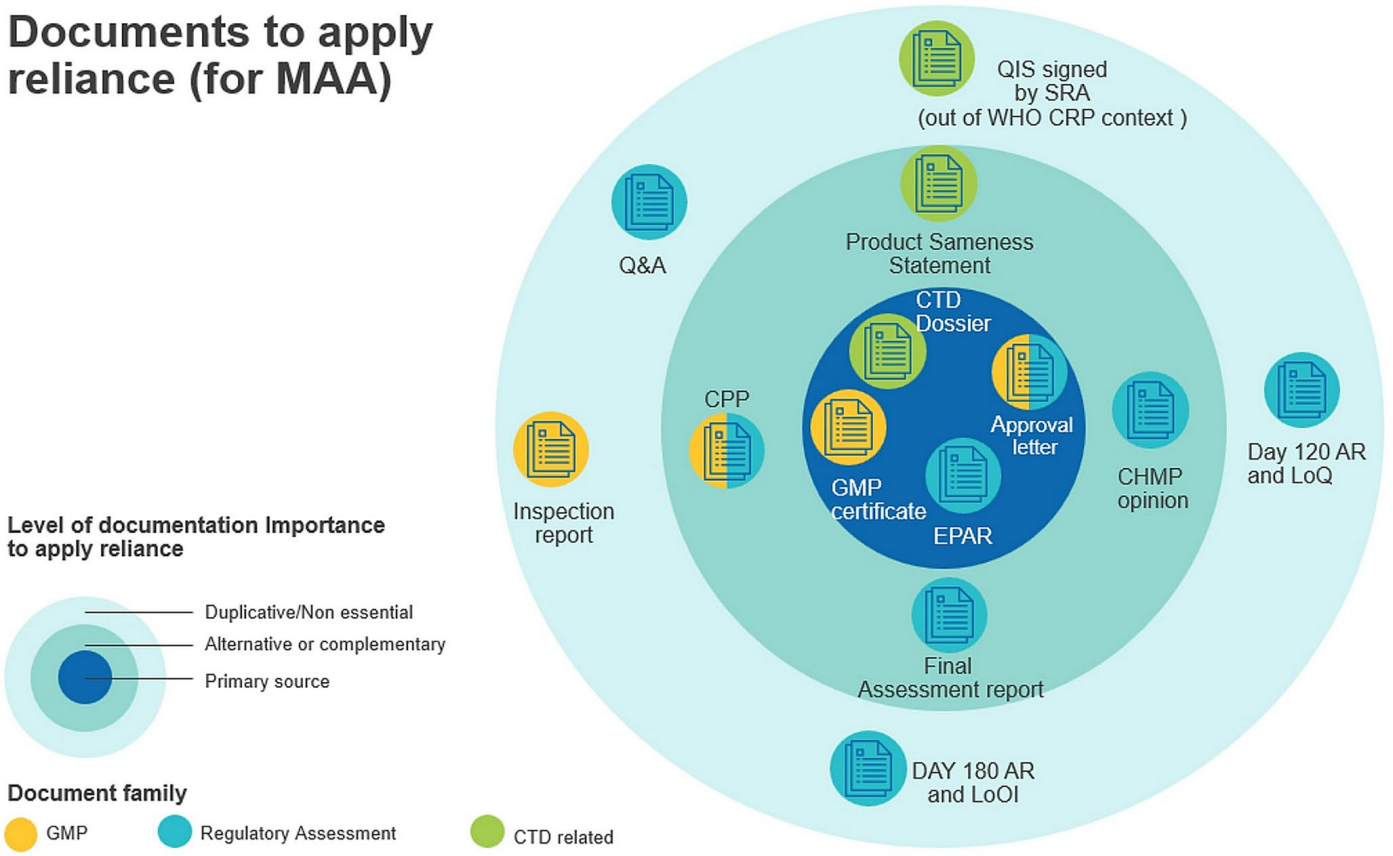
EMA Focus Group recommendations on documents to apply reliance for MAA

Taking into account the lack of consistency and variety of documents (as per Fig. 3), the EMA Focus Group on reliance has developed some recommendations that could help the relying authority streamline the required data to make an informed reliance decision (see Fig. 4). This graphic identifies the documents needed as primary or alternative/complementary sources to facilitate reliance for a MAA, aligning with EMA key messages that were shared during the EMA webinar on 19 March 2024 with more than 1600 participants including 48 regulators attending. Proceedings including Q&As are now available on line [15].

A core set of documents are considered as appropriate tools to apply reliance in addition to the CTD dossier:

- *The GMP certificate confirming GMP Compliance (available in the EudraGMPD data base)*, *approval letter of the marketing authorization (EC decision) and EPAR (European Public Assessment Report) provide a good basis for informed reliance on the work done by EMA.*

They are the primary source of information as they are publicly available and easily accessible on EMA or EC websites, thus do not need further legalization. In particular, the outcome of EMA’s robust review process is captured in the EPAR (which reflects the final CHMP assessment report) even if it does not include details which may be considered commercially confidential information and any personal data.

- *Transparency is an important feature of EMA’s operations*, *and EPARs promote an understanding on what was reviewed and the rationale for decision making*, *promoting confidence and trust.*

Transparency allows public scrutiny and trust, and this is an important feature of EMA’s operations, thus EMA’s decision making is reflected in the public documents. In those cases where the EPAR is not accepted, only the final (CHMP) Assessment Report provided with the CHMP opinion at Day 210 (end of centralized procedure) represents the final scientific discussion on the benefit risk assessment and conclusion of the respective EMA Committees [13].

The EMA recommends in their pre-authorisation guidance for users of the centralised procedure [13] (question 5.1.11), that the MAH/applicant is the one sharing the final (CHMP) Assessment Report with the relying agency. The document states that “when sharing EMA assessment or inspection documents, the MAH/applicant should ensure compliance with the Union legislation on the protection of personal data, including Regulation (EU) 2016/679 [16] and Regulation (EU) 2018/1725. The MAH/applicant assumes any and all liabilities related to any disclosure, particularly with regard to the need to redact certain references in the documents where appropriate or legally needed (e.g. personal data of the assessor/inspector, quality and manufacturing commercial information– note: as per Article 13 alinea 3 of Regulation (EC) 726/2004) [17]. The MAH/applicant should always ensure that the EMA is recognised as the source of the original documents”.

More information on use of assessment reports can be found as well in a recent study conducted by CIRS [18]. This study, which comprised two perception surveys, one with companies and the other one with relying NRAs, focused on evaluating the use and importance of non-public documents (e.g. full assessment reports), as well as public documents (e.g. public assessment reports) during a reliance review.

- *Other documents may be used as alternative or complementary reliance tools*:

The eCPP covers several aspects (registration, GMP compliance and marketing status) as an alternative to the approval letter (EC decision) and GMP certificates per involved manufacturing sites. Of note, the EMA issues an eCPP instead of printed certificates. The EMA online verification tool confirms the validity and authenticity of the eCPP that is increasingly used by countries as a substitute to legalization/apostille.

One important component to apply reliance is trust from the relying authority. To this extent, confirmation of the applicability of the assessment outcomes of the RRA for regulatory decision making by the relying authority is either supported by the same dossier as originally submitted and/or by highlighting any differences in dossier to justify product sameness [1]. In complement to the CTD dossier, it is thus the applicant’s responsibility to highlight and justify any differences compared to the full dossier as originally submitted to allow for a risk-based application [19] of reliance as recommended in WHO good reliance practice [1] (Chap. 5.4). To promote transparency in this space and build trust, IFPMA has proposed a template for description of differences [9] for companies to highlight and justify potential differences helping the relying authority decide how and if to apply reliance (through verification, abridged review) or not (i.e. full assessment pathway). For example, if the manufacturing site has not been assessed by the Reference Agency, then the relying Agency may need to perform an abridged review based on available documentation (e.g. comparability assessment, GMP status…) or take additional steps to decide that the differences have no impact on the quality, efficacy and safety of the physical product.

- *Reliance processes may be adapted as Agencies become more familiar and proficient in risk-based reviews and consider redundancies in the required data to make informed decisions.*

Other documents may be duplicative or useful in the context of capacity building but not required in routine and efficient reliance implementation practice: this is the case for all interim assessment reports (Rapporteur D80, CHMP D120, CHMP D180…) and related answers to questions produced alongside the EMA centralised procedure which represent the status of the evaluation at different timepoints as well as the QIS which is used in the specific context of WHO collaborative procedures [20].

Similarly, GMP certificates confirm the status of compliance of the site at the date of inspection and can be used to apply reliance. EudraGMDP is a public database maintained by the EMA which contains all information on EEA sites having a manufacturing, wholesale, and/or importation license for medicinal products issued by EU/EEA authorities. It also includes Good Manufacturing Practice certificates for EEA sites and third country sites issued after an inspection by either the EEA or MRA (Mutual Recognition Agreement) partners, as well as statements of non-compliance with GMP for EEA and third country sites.

Inspection reports are issued by the lead national/regional inspectorate or by inspectorate in third countries that have performed the inspection and are shared with the manufacturing site(s), inspection team and relevant coordinating inspectorate. For centrally authorized product inspections, these are available within EMA, to EU/EEA inspectorates and MRA (Mutual Recognition Agreement) partners. After any inspection, GMP certificates or respective GMP non-compliance statements are issued. Thus, inspection reports may not be needed in addition to GMP certificates.

## Conclusion

It is encouraging to see the wide use of reliance pathways for marketing authorization, which aim to make more efficient use of the regulatory resources globally. This principle allows leveraging the output of other authorities whenever possible while placing a greater focus at national level on value-added regulatory activities that cannot be undertaken by other authorities. This review demonstrates that leveraging a reference regulatory authority assessment can facilitate decision-making and help accelerate the approval of new MAAs by other regulatory authorities. The decision and process to practice reliance rests with the national regulatory authority.

Through a survey from the EMA Focus Group on reliance, important quantitative and qualitative information on the use of reliance based on EMA could be acquired, sharing further lessons learnt and best practice to facilitate such reliance pathway. According to data gathered from across EU trade associations (EFPIA, Europabio, Vaccines Europe, Eucope and Medicines for Europe), EMA is one of the most frequently selected reference regulatory authority globally due to the detailed decision making reflected on its public assessment report (EPAR), ease of access, confidentiality and transparency in the reliance tools generated during the centralised procedure.

Flexibility and agility are key for the smart implementation of reliance pathways. It is thus important to measure the impact of these activities, to adjust and focus on opportunities and avoid redundancies in requirements. Engagement of all stakeholders can help prioritize and converge on core regulatory documentation to implement “informed reliance” pathways.

Moving forward, extending the scope of reliance to other areas in need (post-approval changes [21, 22]clinical trials oversight [1]regulatory inspection [1]lot release/import and laboratory testing [1, 23]) will bring further efficiencies within the regulatory ecosystem. Promising results have been shared in the latest ICMRA PQKM (Pharmaceutical Quality Knowledge Management) reports [24, 25] as well as illustrated in recent pilots using reliance for inspections [26, 27] and post approval changes [28, 29].

Accelerating timelines for approval is one of the key components of reliance pathways but other important aspects to be considered include promoting alignment of requirements, capacity building and collaboration between authorities and ultimately improving access to quality-assured medical products and potentially reducing the risk of shortages when applied to post-approval changes.

To fully realize the benefits of regulatory reliance, using the appropriate documentation generated alongside the EMA centralised procedure will help get innovative products to patients sooner as well as reduce redundancies and regulatory burden for both health authorities and industry.

## Electronic Supplementary Material

Below is the link to the electronic supplementary material.

Supplementary Material 1

Supplementary Material 2

## Data Availability

No datasets were generated or analysed during the current study.
