# Supplementary material for: Reliance into Action: Understanding EMA Documents to Streamline Reliance for Marketing Authorization Applications
Source: Ther Innov Regul Sci. 2025 Jun 28;59(5):1032–41. doi: 10.1007/s43441-025-00824-9 (PMC12446411; doi:10.1007/s43441-025-00824-9)
Supplement: Supplementary file 1 — Supplementary Material 1 [file 43441_2025_824_MOESM1_ESM.docx]

**Introduction:**

More than ever, developing regulatory processes which enable fast and equitable access to medical products to all populations across the world is of utmost importance. One of those regulatory processes is **Reliance**, defined by WHO (TRS 1033, Annex 10) as follows: “The act whereby the regulatory authority in one jurisdiction takes into account and gives significant weight to assessments performed by another regulatory authority or trusted institution, or to any other authoritative information, in reaching its own decision.”

EMA is willing to help in facilitating the implementation of reliance processes, once they better understand about the current issues which need to be addressed. This survey is launched across the pharmaceutical industry to identify key issues and opportunities for improvement. The survey also aims to understand the use of EMA as reference agency in unilateral^[[1]](#footnote-2)^ reliance pathways to further feed subsequent discussions with EMA Focus Group.

When filling out this survey, please consider only reliance for marketing authorization/post-approval changes. GMP and QC testing are out of scope.

**Please only send one response per company**

| **Question**  **(numbers will be redefined once survey is set up)** | **Response options** | **Question dependencies / Comments** |
| --- | --- | --- |
| **Group A – Reliance on EMA** | | |
| 1. In the last 2 years, has your company used unilateral reliance procedures with EMA as a reference agency (for regulatory assessment purpose) for any of the submissions in focus? | Yes /No | If “No”, skip to Group B |
| 1. In the last 2 years, for which of the following non EU/EEA countries has your company used unilateral reliance procedures with EMA as a reference agency (for regulatory assessment purpose)?   Fill out all that apply | Use list of countries of the world^[[2]](#footnote-3)^ | [List of countries.xlsx](https://egagenerics-my.sharepoint.com/:x:/g/personal/salmeida_medicinesforeurope_com/EflstHkwDd1AuSE83RAW1usBz5QA86oS3rdlUh2CYnwHEQ?e=IACXsA) |
| 1. Used for Initial application? | Yes/No | Per each country |
| 1. Used for CMC post-approval change | Yes/No | Per each country |
| 1. Used for new indication extension or labelling post approval changes | Yes/No | Per each country |
| 1. Did you **have to** provide a justification or statement with regards to product sameness? | Yes/No/It depends | Per each country |
| 1. Did you provide the same dossier (CTD M2-5) or variation package to the reference agency and relying agency? | Yes/No/It depends | Per each country |
| 1. In the last 2 years, for each of the countries where your company used unilateral reliance procedures, which information did your company provide from the reference agency? Select all that apply | Each option (Yes/No)  Approval Letter  eCPP  EPAR (European Public Assessment Report)  Unredacted EMA Final CHMP assessment report (before publication)  Day 180 Assessment report + LoQ  Day 120 Assessment report + LoQ  GMP certificate  Inspection report  Questions & Answers  Quality Information Summary (QIS) signed by SRA  Other | Per each country |
| **Group B – Reliance on EU National Competent Authorities** | | |
| 1. In the last 2 years, has your company used unilateral reliance procedures with EU National Competent Authorithy(ies) as a reference agency (for regulatory assessment purpose)? | Yes  No | If No, skip to Group C |
| 1. Which was/were the reference authority(ies)? | Free text |  |
| 1. What was positive and what were the challenges with EU NCA as reference agency? | Free text |  |
| **Group C – General Questions** | | |
| 1. From your company’s perspective, what are the benefits from using reliance (please rank the items) | - Reduction of timelines to approval - Predictable review / approval timelines - Capacity building (review and/or resources) amongst regulators - Reduction of number of questions from the relying agency - Aligned PI (product information) - Perceived reduced resource requirements for industry - reduction of country specific requirements and/or harmonization with SRA - Other (please specify) | General |
| 1. What are the challenges your company has experienced using reliance (please rank the items) | - No clear understanding of reliance definition - No regulatory framework to support a reliance submission - No clear guideline(s)/guidance with requirements - No Confidentiality Agreement/Memorandum of Understanding between SRA and NRA - Required unredacted assessment report - Not enough SRAs or WLA-listed agencies to apply reliance - Strict interpretation of product sameness prevented reliance - Additional administrative requirements/documents including local M1 and other local documents - Reliance not practiced (second review performed instead of verification or abridged review focusing on specific local aspects) - No acceleration of the timelines - Restricted scope i.e. no application to other areas (specific molecules only, LCM, QC testing...) - Difficulties accepting eCPP - Long submission lag time - Other (please specify) | General |
| 1. If you could change one thing that would result in a reduction of regulatory burden and offer an opportunity for faster and more predictable approvals what could it be? | Free text |  |
| 1. Any suggestion on what could EMA do to support reliance? | Free text | General |
| 1. Add any additional comments you may have specifically if you selected the answer “it depends” or “other” in previous questions | Free text | General |

1. Reliance may be unilateral, for example, when a country chooses to rely on an assessment from another country unilaterally and without reciprocity (WHO Good Reliance Gdl 2020). There is likely no further communication or interaction between the countries. [↑](#footnote-ref-2)
2. https://www.itu.int/en/ITU-D/Statistics/Pages/definitions/regions.aspx [↑](#footnote-ref-3)
